# Extraction with Acidified Methanol—An Easy and Effective Method of Methyl Chlorogenate Formation, as Studied by ESI-MS

**DOI:** 10.3390/molecules27217543

**Published:** 2022-11-03

**Authors:** Karolina Szymborska, Rafał Frański, Monika Beszterda-Buszczak

**Affiliations:** 1 Faculty of Chemistry, Adam Mickiewicz University, Uniwersytetu Poznańskiego 8, 61-614 Poznań, Poland; 2 Department of Food Biochemistry and Analysis, Poznań University of Life Sciences, Mazowiecka 48, 60-623 Poznań, Poland

**Keywords:** *Crataegus monogyna*, extraction, liquid chromatography, mass spectrometry, methyl chlorogenate, chlorogenic acids, flavonoids

## Abstract

Among the different Hawthorn species, *Crataegus monogyna* seems to be one of the most often used in herbal medicine, and is commercially available. The methanolic extract and the acidified methanol extract of an herbal medicinal product based on *Crataegus monogyna* inflorescences were analyzed by using high-pressure liquid chromatography/electrospray ionization mass spectrometry (HPLC/ESI-MS). On the basis of *m/z* of [M-H]^−^ ions and characteristic fragmentation patterns, a number of polyphenolic compounds, namely flavonoids and chlorogenic acids, were identified. It was found that the contents of the acid extract were enriched with methyl chlorogenates showing attractive biochemical properties. Analogous results were obtained for other plant materials, e.g., nectarine kernels. Apart from that, acid extraction had a minor influence on the polyphenolic compounds present in the plants, and thus it did not affect the natural antioxidant values of the plant extracts.

## 1. Introduction

Chlorogenic acid is one of the most important compounds belonging to the large family of esters of cinnamic acid derivatives (mainly hydroxyl derivatives) and quinic acid, present in a vast number of plants. Formally, chlorogenic acid is *trans*-5-*O*-caffeoylquinic acid (although the naming of chlorogenic acids is disputable [1]), as the term chlorogenic acids may also refer to the isomers of *trans*-5-*O*-caffeoylquinic acid and to the esters of other derivatives of cinnamic acid (e.g., coumaric acid) with quinic acid. The biological activities and extraction procedures of chlorogenic acids have been widely studied for years and have been summarized in a number of review papers [1,2,3,4,5,6,7].

**Scheme 1:**
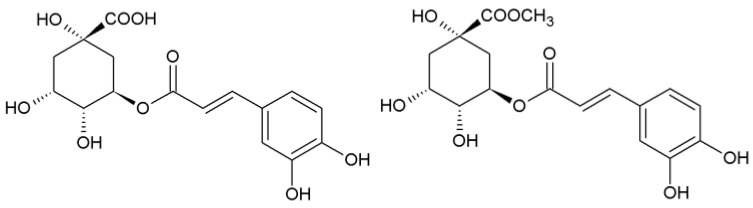
Structure of chlorogenic acid (*trans*-5-*O*-caffeoylquinic acid) and methyl chlorogenate.

Methyl ester of chlorogenic acid (methyl chlorogenate, Scheme 1) is a very interesting compound occurring in plants and showing biological activity, e.g., as an antioxidant, although it is much less commonly used than chlorogenic acid.

Methyl chlorogenate shows strong radical scavenging ability towards the 2,2′-diphenyl-1-picrylhydrazyl radical (DPPH), superoxide scavenging activity and hydroxyl radical scavenging activity [8,9,10,11]. Methyl chlorogenate is a potential candidate to be developed as an effective anti-inflammatory agent [12]. Furthermore, it has been found to be a very effective intermediate in the chemo-enzymatic route of synthesis of chlorogenate fatty esters [13]. The obtained fatty esters show antifungal activity and may have strong antioxidant properties in emulsion or in oil, as discussed in detail elsewhere [14,15,16,17].

In this study, it is demonstrated that methylation of chlorogenic acids present in plant material can be effectively performed by extraction using acidified methanol. The plant material was *Crataegus monogyna* inflorescences purchased from a local pharmacy. Among the different Hawthorn species, *Crataegus monogyna* seems to be one of the most often used in herbal medicine, is commercially available. It has been demonstrated that, in order to obtain the required properties of Hawthorn products, the extraction technique used is of crucial importance [18,19,20,21].

## 2. Results and Discussion

**Figure 1:**
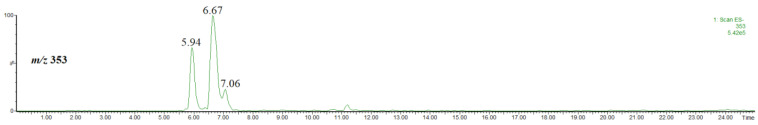
Single ion chromatogram of the ion at *m/z* 353, thus the [M-H]^−^ ion of chlorogenic acid and its isomers.

At first we identified the chlorogenic acids present in the used plant material. For this purpose, we performed HPLC/ESI-MS analysis of the extract by using methanol (not acidified methanol). Figure 1 shows the single ion chromatogram of the ion at *m/z* 353, identified as the [M-H]^−^ ion of chlorogenic acid and its isomers.

**Figure 2:**
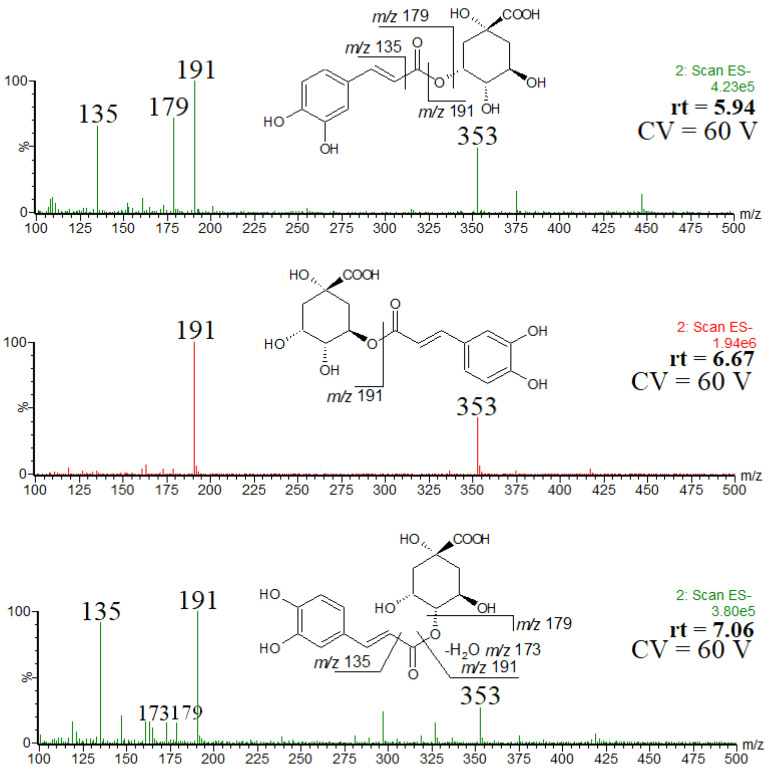
ESI mass spectra of chlorogenic acid and its isomers, and the observed fragmentation patterns.

Taking into account the intensity of chromatographic peaks and the elution order, the isomers were identified as *trans*-3-*O*-caffeoylquinic acid (neochlorogenic acid) (rt = 5.94), *trans*-5-*O*-caffeoylquinic acid (rt = 6.67) and *trans*-4-*O-*caffeoylquinic acid (cryptochlorogenic acid) (rt = 7.06 min) [7,22]. Furthermore, a comparison of the spectra obtained at a higher cone voltage, shown in Figure 2, also confirmed the elution order. The fragmentation patterns recorded for each isomer (Figure 2) are in agreement with the results reported elsewhere (although it is obvious that the relative ion abundances may be different) [23,24]. Namely, the abundant product ion at *m/z* 191 and the lack of other product ions indicates the presence of trans-5-*O*-caffeoylquinic acid, the abundant product ion at *m/z* 179 indicates the presence of *trans*-3-*O*-caffeoylquinic acid and the product ion at *m/z* 173 indicates the presence of *trans*-4-*O*-caffeoylquinic acid. Therefore, although in a number of papers devoted to phenolic constituents of *Crataegus monogyna* only one isomer of chlorogenic acid has been mentioned [25,26,27,28,29], the presence of the above three regioisomers seems to be irrefutable [22]. To the best of our knowledge, only in one paper the geometric isomer, namely *cis*-3-*O*-caffeoylquinic acid, has been tentatively identified as a minor constituent of *Crataegus monogyna* [30]. Furthermore, the geometric isomers have very similar mass spectra [24], whereas the spectra obtained in our study are substantially different (Figure 2).

**Figure 3:**
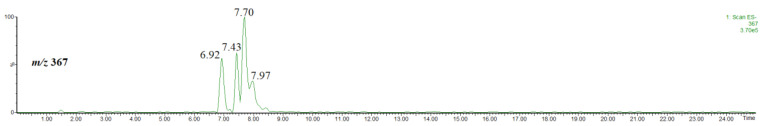
Single ion chromatogram of the ion at *m/z* 367, thus the [M-H]^−^ ion of methyl chlorogenate and its isomers.

**Figure 4:**
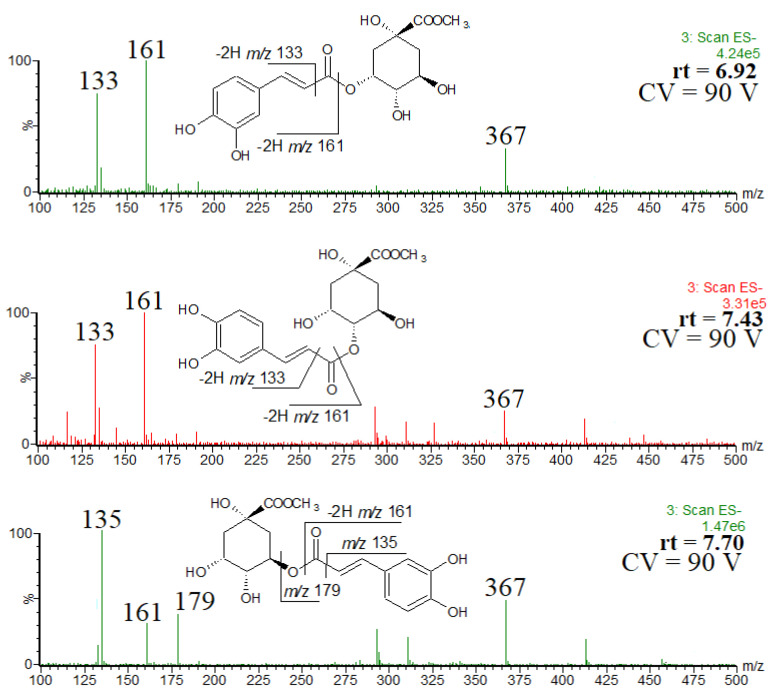
ESI mass spectra of methyl chlorogenate and its isomers, and the observed fragmentation patterns.

In the extract obtained by using acidified methanol, methyl chlorogenate and its isomers were detected. Figure 3 shows the single ion chromatogram of the ion at *m/z* 367, identified as the [M-H]^−^ ion of methyl chlorogenate and its isomers. On the basis of the elution order and observed fragmentation patterns (Figure 4), the compounds were identified as methyl 3-*O*-caffeoylquinate (rt = 6.92 min), methyl 4-*O*-caffeoylquinate (rt = 7.43 min) and methyl 5-*O*-caffeoylquinate (rt = 7.70 min) [31,32]. The abundant product ion at *m/z* 179 indicates the presence of 5-*O*-caffeoylquinate. Although 3-*O*-caffeoylquinate and methyl 4-*O*-caffeoylquinate had very similar spectra, they were differentiated on the basis of the elution order [31,32].

In other words, for each of the chlorogenic acids identified in the methanol extract, its methyl ester was found in the acidified methanol extract. More difficult was to rationalize the minor fourth peak at rt = 7.97 min. It may be that during the extraction in acidic conditions we deal with the formation of low abundant by-products, as a result of *trans/cis* isomerization [33,34]. However, it was difficult to prove unequivocally, therefore, the spectrum of the minor by-product is not shown and discussed here. Of course, we are aware that the occurrence of *trans/cis* isomerization may affect the desirable properties of methyl chlorogenates. On the other hand, to the best of our knowledge, the differences in biochemical properties of methyl chlorogenate *trans* and *cis* isomers are not known, therefore, a positive effect of the *trans/cis* isomerization cannot be excluded.

**Figure 5:**
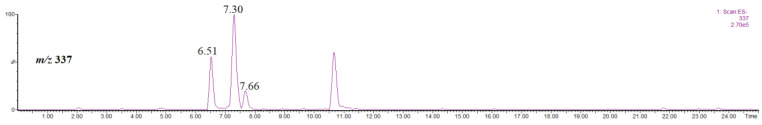
Single ion chromatogram of the ion at *m/z* 337, thus the [M-H]^−^ ion of coumaroylquinic acid isomers.

Figure 5 shows the single ion chromatogram of the ion at *m/z* 337, identified as the [M-H]^−^ ion of coumaroylquinic acid isomers.

Two major compounds were detected at rt = 6.51 min and rt = 7.30 min, besides a minor one at rt = 7.66 min (Figure 5). Some of the published data concerning the occurrence of the coumaroylquinic acid isomers in the *Crataegus monogyna* suggest the existence of geometric isomers (namely *trans/cis*-5-*O*-coumaroylquinic acids) [29,35], while the other data suggest the existence of regioisomers (*trans*-3-, 4-, 5-*O*-coumaroylquinic acids) [22]. 

**Figure 6:**
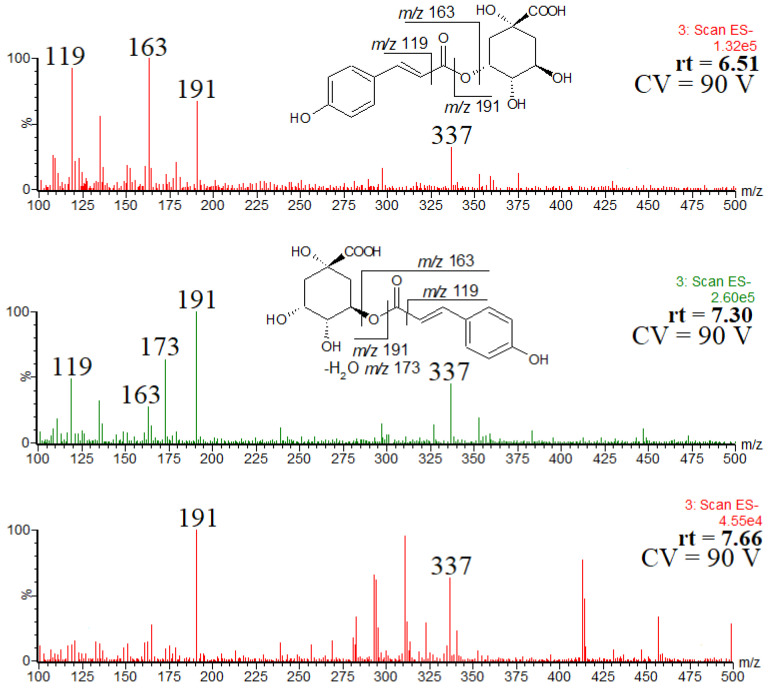
ESI mass spectra of coumaroylquinic acid isomers, and the observed fragmentation patterns of the two main isomers.

Figure 6 shows the spectra of the detected isomers. Since the observed fragmentation patterns are substantially different, it is reasonable to conclude that the compounds are regioisomers.

The isomer eluted first was identified as *trans*-3-*O*-coumaroylquinic acid, as indicated by the abundant product ion at *m/z* 163. The second isomer was identified as *trans*-5-*O*-coumaroylquinic acid, as indicated by the abundant product ion at *m/z* 191 [23]. The presence of these two main regioisomers is in good agreement with the data reported by Kuczkowiak et al. [22]. Furthermore, the obtained UV spectra confirmed the presence of *trans* isomers, as indicated by the absorption maxima (supplementary materials, Figure S1) [36]. It was difficult to prove unequivocally the structure of the third isomer, namely the minor one at rt = 7.66 min (the peak at rt = 10.7, Figure 5, does not correspond to the coumaroylquinic acid, as indicated by its retention time).

**Figure 7:**
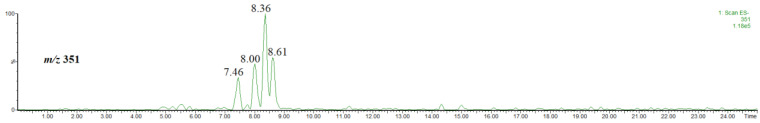
Single ion chromatogram of the ion at *m/z* 351, thus the [M-H]^−^ ion of methyl coumaroylquinates.

Figure 7 shows a single ion chromatogram of the ion at *m/z* 351, thus the [M-H]^−^ ion of methyl coumaroylquinates obtained for the extract prepared by using acidified methanol. As clearly seen in Figure 7, four isomers were detected.

**Figure 8:**
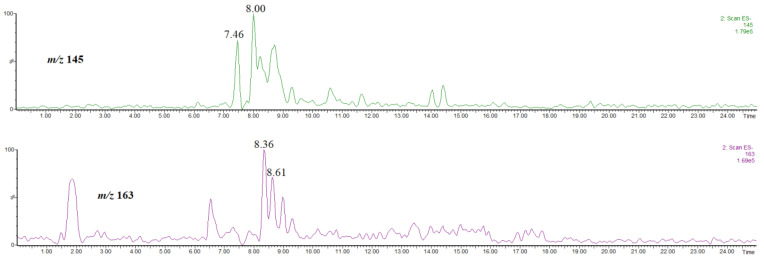
Single ion chromatograms of the ion at *m/z* 145 and 163, thus the product ions of methyl coumaroylquinates.

The abundances of the isomers are much lower than those of the other compounds discussed above. Furthermore, the isomers co-eluate with the other abundant compounds present in the extract, e.g., that at rt = 7.46 co-eluates with methyl 4-*O*-caffeoylquinate (rt = 7.43 min, Figure 3), while that at rt = 8.00 co-eluates with hyperoside (rt = 8.06 min, supplementary material, Figure S3). Therefore, an unambiguous isomer identification on the basis of the obtained mass spectra was difficult. On the other hand, it has been already established that the product ion at *m/z* 145 is characteristic of methyl 3-, 4-*O*-coumaroylquinate, whereas that at *m/z* 163 is characteristic of methyl 5-*O*-coumaroylquinate [31]. Figure 8 shows the single ion chromatograms of the ions at *m/z* 145 and 163. On the basis of a comparison of Figure 7 and Figure 8, it is evident that the signal at *m/z* 145 corresponds to the product ion of the isomer at rt = 7.46 and 8.00 min, whereas that at *m/z* 163 corresponds to the product ion of the isomer at rt = 8.36 and 8.61 min.

Since in the methanol extract two main isomers were detected, *trans*-3-*O*-coumaroylquinic acid and *trans*-5-*O*-coumaroylquinic acid (Figure 5 and Figure 6), it is reasonable to conclude that in the extract prepared by using acidified methanol there are methyl *cis/trans*-3-*O*-coumaroylquinate and methyl *cis/trans*-5-*O*-coumaroylquinate.

**Figure 9:**
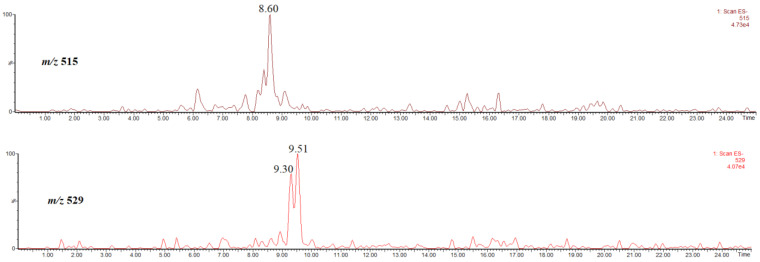
Single ion chromatogram of the ion at *m/z* 515, thus the [M-H]^−^ ion of 3,5-di-*O*-caffeoylquinic acid and single ion chromatogram of the ion at *m/z* 529, thus the [M-H]^−^ ion of methyl 3,5-*O*-dicaffeoylquinates.

Figure 9 shows the single ion chromatogram of the ion at *m/z* 515, identified as the [M-H]^−^ ion of, most probably, 3,5-di-*O*-caffeoylquinic acid [22], obtained in the extract prepared by using methanol, and the single ion chromatogram of the ion at *m/z* 529, thus, the [M-H]^−^ ion of methyl 3,5-di-*O*-caffeoylquinates, obtained in the extract prepared by using acidified methanol. The abundances of these compounds are low, therefore their spectra are not shown/discussed here.

It should be stressed that in the extracts obtained by using acidified methanol, the chlorogenic acids were also detected; of course, their abundances were different than those in the extracts obtained by using methanol (an exemplary chromatogram is shown in the supplementary material, Figure S2). In other words, the acid extraction does not eliminate the chlorogenic acids, but yields additional compounds, namely methyl chlorogenates.

In both analyzed extracts we also detected flavonoid compounds, characteristic of *Crataegus monogyna*, e.g., hyperoside, vitexin-2′′-*O*-rhamnoside, quercetin (the latter only in the acid extract due to the partial hydrolysis of quercetin glycosides, [37,38]). Their identification was supported by the characteristic product ions [39,40,41], as shown in the supplementary material, Figures S3–S5. The only flavonoid compound which was not detected in the acid extract was 4′′′-acetylvitexin-2′′-*O*-rhamnoside, as it had lost an acetyl group in the acid solution. This compound is a relevant chemo-taxonomic marker for *Crataegus monogyna* and, of course, was detected in the methanolic extract [38]. It is also worth adding that methylation of flavonoid compounds did not occur during the acidified extraction. 

The above discussion concerns the results obtained for the extract of *Crataegus monogyna*. However, it should be pointed out that similar results have been obtained for a number of other plant materials. As an example, the chromatograms of the extracts of kernels of *Prunus persica* var. *nucipersica* (nectarine) are shown in the supplementary material (Figures S6–S8). It is worth adding that the extract of nectarine kernels may have interesting biochemical properties as discussed in detail elsewhere [42,43].

## 3. Materials and Methods

### 3.1. Preparation of the Analyzed Extracts

The *Crataegus monogyna* inflorescences (herbal medicinal products supporting the work of the heart and circulatory system) were purchased from a local pharmacy. A portion of 2 g was extracted with 10 mL of pure methanol or with 5% methanolic solution of hydrochloric acid (acidified extraction), the sample was shaken at 500 rpm for 30 min (Vortex 3, IKA-Werke GmbH, Staufen im Breisgau, Germany), sonicated and filtered through syringe filters with a pore size of 0.45 μm (Macherey-Nagel GmbH, Düren, Germany). Prior to the HPLC/ESI-MS analysis, the sample was further diluted at 1:1 in pure methanol (stored at 5 °C). Analogous procedures were applied in order to prepare the extracts from other plant materials, e.g., from nectarine kernels.

### 3.2. Performance of the HPLC/MS and HPLC/UV Analyses

HPLC/ESI-MS analyses were performed using a Waters model 2690 HPLC pump (Milford, MA, USA), a Waters/Micromass ZQ2000 mass spectrometer (single quadrupole type instrument equipped with electrospray ion source, Z-spray, Manchester, UK). The software used was MassLynx V3.5 (Manchester, UK). Using an autosampler, the sample solutions were injected onto the C18 Atlantis T3 column (3 µm, 100 mm × 3 mm i.d.). The injection volume was 5 µL. The solutions were analyzed by using a linear gradient of CH_3_CN-H_2_O with a flow rate of 0.4 mL/min. The gradient started from 0% CH_3_CN–95% H_2_O with 5% of a 10% solution of formic acid in water (thus, the formic acid concentration in the mobile phase was 0.5%), reaching 95% CH_3_CN after 15 min, and the latter concentration was maintained for 10 min. The ESI mass spectra were recorded in the *m/z* range 100–1000, in both positive and negative ion mode. However, for analysis of chlorogenic acids and methyl chlorogenates only the data obtained in negative ion mode were found to be useful. In the supplementary material (Figure S9) the total ion current chromatograms obtained for the analyzed extracts of *Crataegus monogyna* inflorescences are shown. The nebulizing and desolvation gas was nitrogen at the flow rates of 100 and 300 L/h, respectively. The source temperature was 120 °C, and the desolvation temperature 300 °C. The electrospray source potentials were: capillary 3 kV, lens 0.5 kV, extractor 4 V and cone voltage 30, 60 and 90 V. It is known that cone voltage (CV) has the greatest impact on the mass spectra recorded. An increase in this parameter leads to the so-called “in-source” fragmentation/dissociation, but too low a cone voltage may cause a decrease in sensitivity. In order to confirm that the detected product ions originate from the respective parent ions ([M-H]^−^ ions), the retention times of the former were checked and compared with the retention times of the latter (e.g., Figure 8 and Figure S10 in the supplementary material).

Waters 996 Photodiode Array Detector was used in order to perform HPLC/UV analysis, in the range 210–600 nm. Chromatographic conditions were identical to those used for HPLC/MS analysis.

## 4. Triple Prime

Summing up, it was clearly demonstrated that by using acidified methanol as an extraction agent, the extracts obtained from the plant material used contained methyl chlorogenates formed as a result of the esterification of chlorogenic acids (methyl chlorogenates were not detected in the methanol extracts and it is well known that the presence of acid speeds up the esterification process). The obtained methyl chlorogenates were identified by HPLC/ESI-MS in negative ion mode on the basis of *m/z* of [M-H]^−^ ions and characteristic fragmentation patterns observed. Almost all polyphenolic compounds, namely flavonoid glycosides and chlorogenic acids, which were present in the analyzed plant material and as a consequence in the methanolic extract, were also found in the extract obtained by using acidified methanol. Of course, in the acid extract, due to the partial hydrolysis of O-glycosidic bond, free aglycone may be also present. The only flavonoid compound which was not detected in the acid extract was 4′′′-acetylvitexin-2′′-*O*-rhamnoside as it had lost an acetyl group in the acid solution. Therefore, extraction with acidified methanol may be regarded as an effective method of enrichment of plant extracts with methyl chlorogenates—promising compounds of interesting biochemical properties.

## Data Availability

Not applicable.

## Supplementary Materials

The following supporting information can be downloaded at: https://www.mdpi.com/article/10.3390/molecules27217543/s1, Figure S1: UV spectra, obtained during HPLC/UV analysis, of the main caffeoylquinic acids and coumaroylquinic acids detected. Figure S2: Single ion chromatogram of the ion at *m/z* 353 obtained for the extract prepared by using acidified methanol. Figure S3: Identification of hyperoside in the methanolic extract of *Crataegus monogyna*. Figure S4: Identification of vitexin-2′′-*O*-rhamnoside in the methanolic extract of *Crataegus monogyna*. Figure S5: Identification of querctein in the acidified methanolic extract of *Crataegus monogyna*. Figure S6: Single ion chromatogram of the ion at *m/z* 337 (the [M-H]^−^ ion of coumaroylquinic acid) and *m/z* 351 (the [M-H]^−^ ion of methyl coumaroylquinate) obtained for the extracts of kernels of *Prunus persica* var. *nucipersica* (nectarine). Figure S7: Single ion chromatogram of the ion at *m/z* 353 (the [M-H]^−^ ion of chlorogenic acid) and *m/z* 367 (the [M-H]^−^ ion of methyl chlorogenate) obtained for the extracts of kernels of *Prunus persica* var. *nucipersica* (nectarine). Figure S8: Single ion chromatogram of the ion at *m/z* 515 (the [M-H]^−^ ion of 3,5-di-*O*-caffeoylquinic acid) and *m/z* 529 (the [M-H]^−^ ion of methyl 3,5-*O*-dicaffeoylquinates) obtained for the extracts of kernels of *Prunus persica* var. *nucipersica* (nectarine). Figure S9: Total ion current chromatograms obtained for the extracts of *Crataegus monogyna* inflorescences by using methanol (top) and acidified methanol (bottom). Figure S10: Single ion chromatogram of the ion at *m/z* 133.
