# Supplementary material for: Extraction with Acidified Methanol—An Easy and Effective Method of Methyl Chlorogenate Formation, as Studied by ESI-MS
Source: Molecules. 2022 Nov 3;27(21):7543. doi: 10.3390/molecules27217543 (PMC9654720; doi:10.3390/molecules27217543)
Supplement: Supplementary file 1 [file molecules-27-07543-s001.zip › molecules-1996323-supplementary.pdf]

Extraction with acidified methanol - an easy and effective method of methyl chlorogenate formation, as studied by ESI-MS – **supplementary material**

Karolina Szymborska[1], Rafał Frański[1,*], Monika Beszterda-Buszczak[2]

[1] - Adam Mickiewicz University, Faculty of Chemistry, Uniwersytetu Poznańskiego 8, 61-614 Poznań, Poland

[2] - Poznań University of Life Sciences, Department of Food Biochemistry and Analysis, Mazowiecka 48, 60-623 Poznań, Poland.

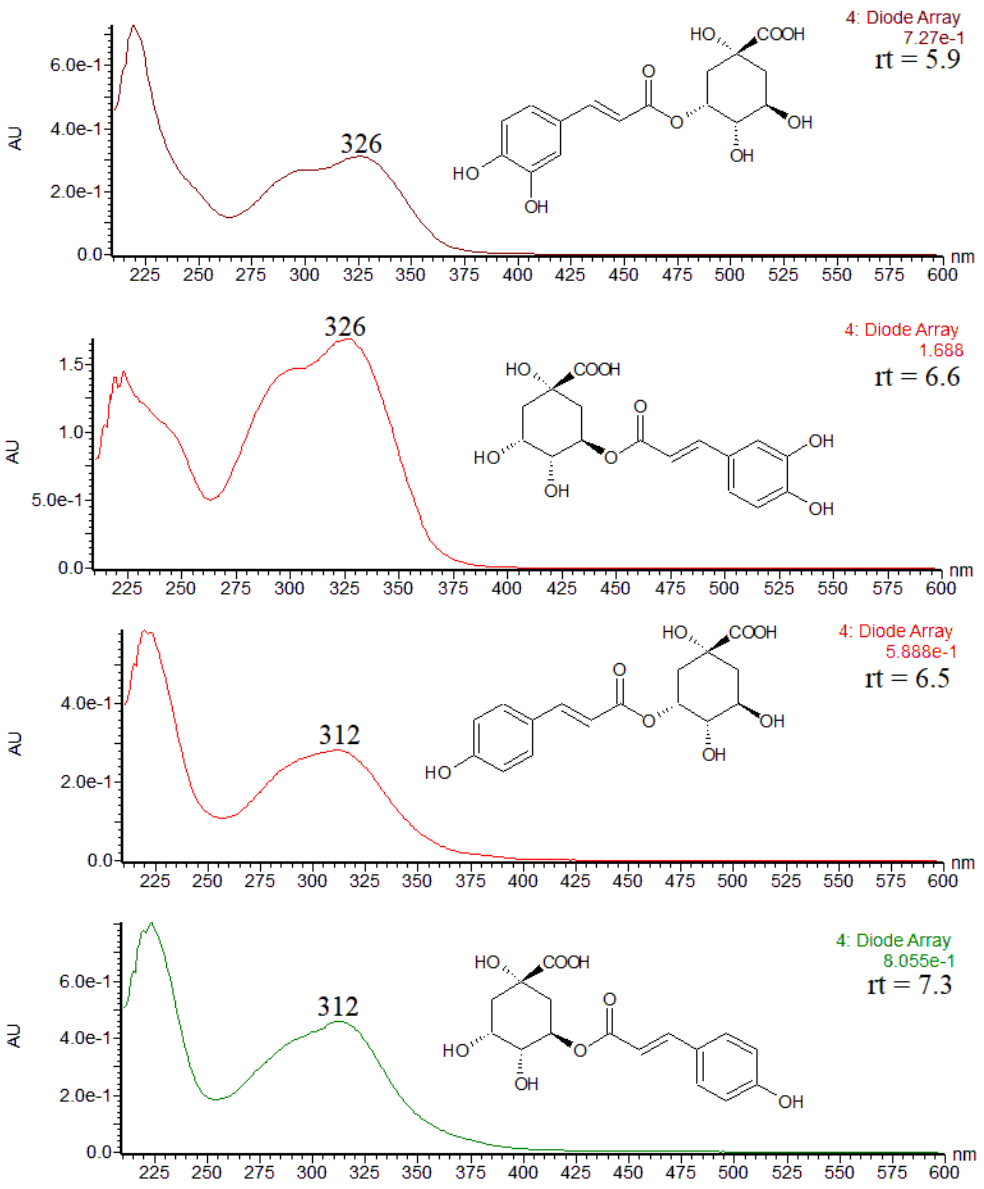

Figure S1. UV spectra, obtained during HPLC/UV analysis, of the main caffeoylquinic acids and coumaroylquinic acids detected.

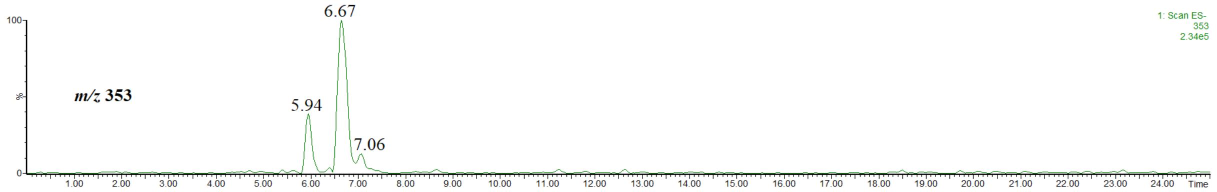

Figure S2. Single ion chromatogram of the ion at *m/z* 353 obtained for the extract prepared by using acidified methanol.

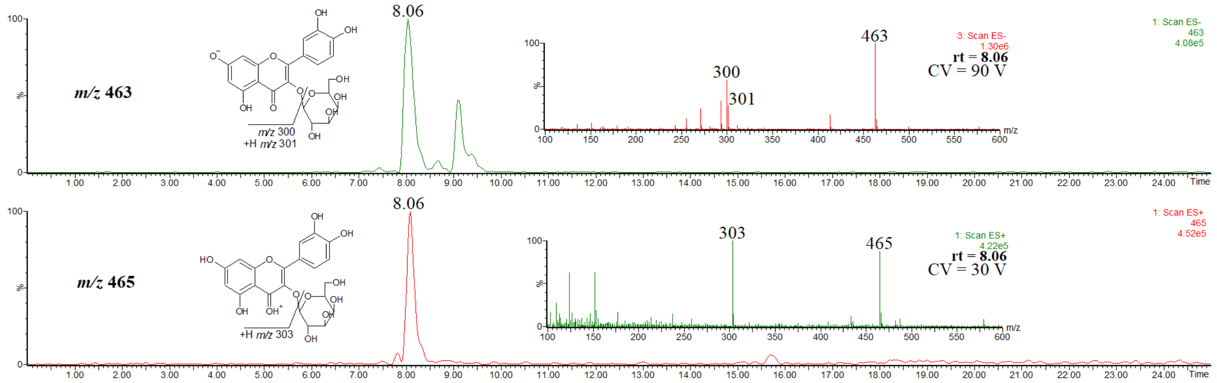

Figure S3. Identification of hyperoside in the methanolic extract of *Crataegus monogyna* ([M-H]$^-$ *m/z* 463, [M+H]$^+$ *m/z* 465).

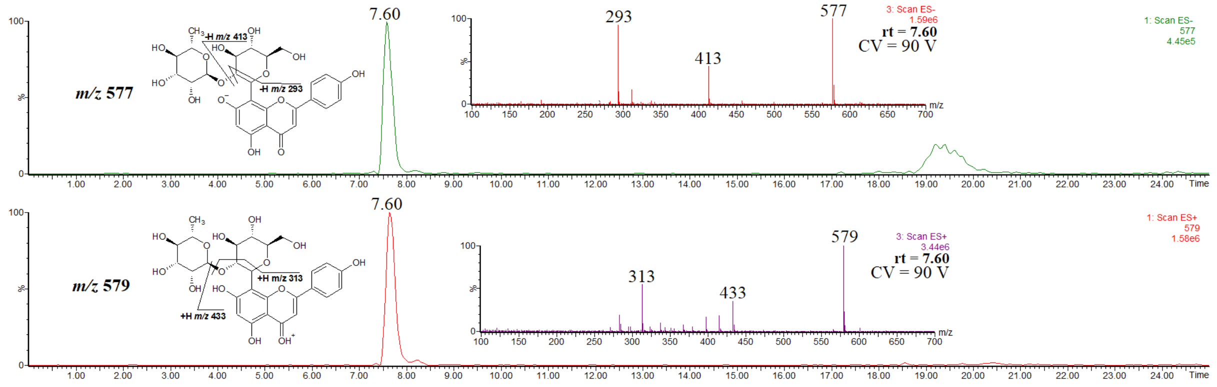

Figure S4. Identification of vitexin-2’’-*O*-rhamnoside in the methanolic extract of *Crataegus monogyna* ([M-H]$^-$ *m/z* 577, [M+H]$^+$ *m/z* 579).

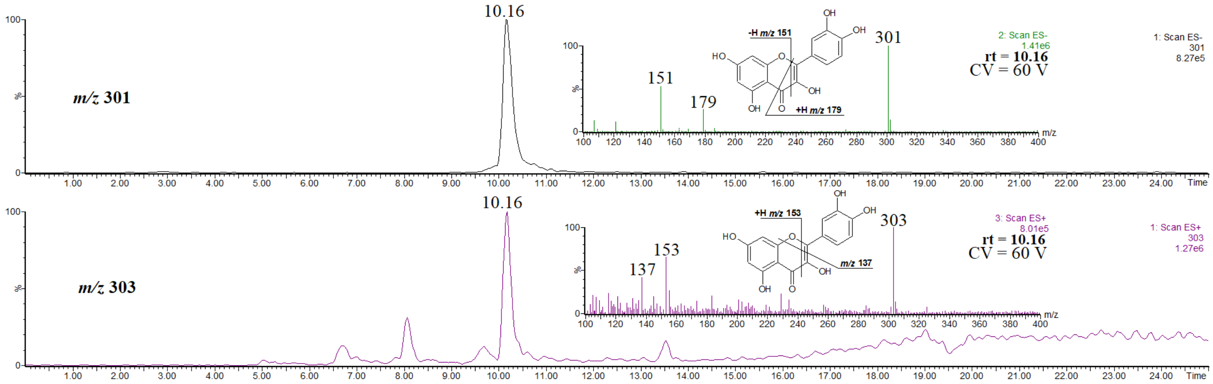

Figure S5. Identification of quercetin in the acidified methanolic extract of *Crataegus monogyna* ([M-H]$^-$ *m/z* 301, [M+H]$^+$ *m/z* 303).

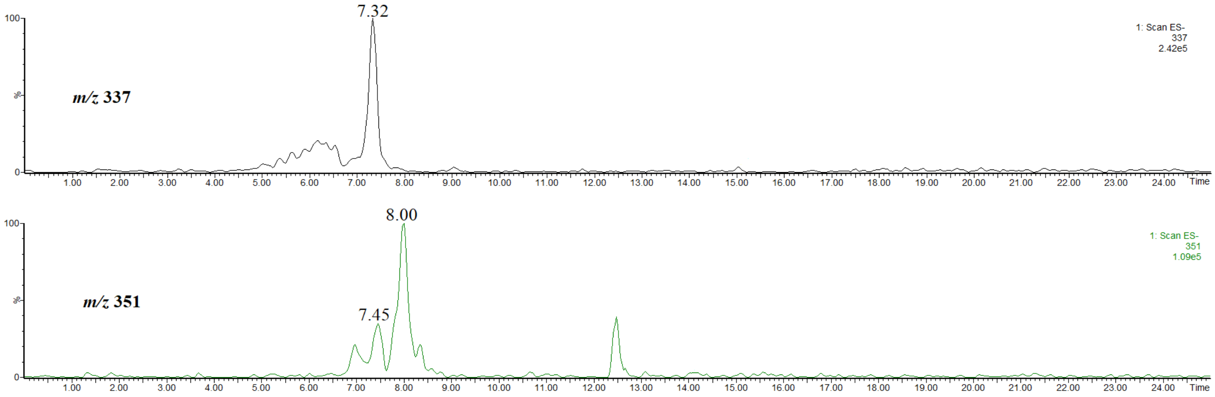

Figure S6. Single ion chromatogram of the ion at *m/z* 337 (the [M-H]$^-$ ion of coumaroylquinic acid) and *m/z* 351 (the [M-H]$^-$ ion of methyl coumaroylquinate) obtained for the extracts of kernels of *Prunus persica* var. *nucipersica* (nectarine).

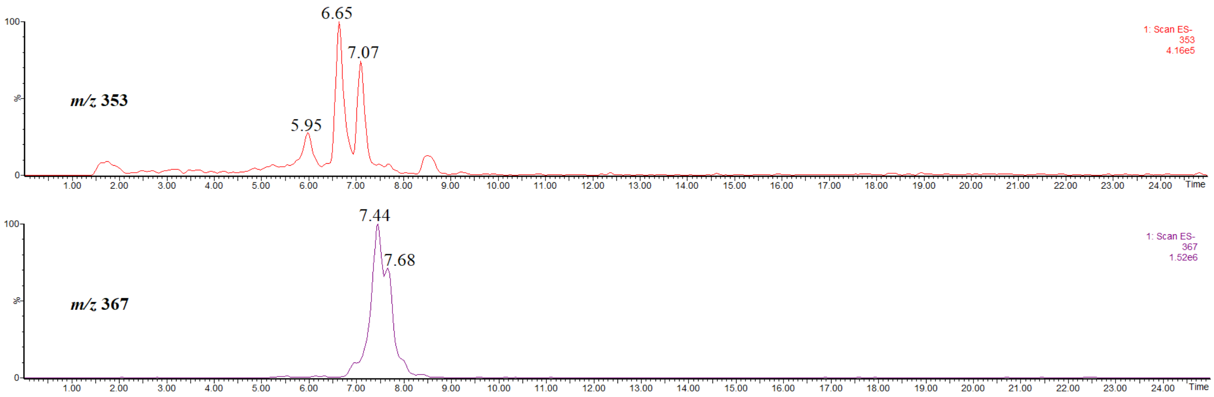

Figure S7. Single ion chromatogram of the ion at *m/z* 353 (the [M-H]$^-$ ion of chlorogenic acid) and *m/z* 367 (the [M-H]$^-$ ion of methyl chlorogenate) obtained for the extracts of kernels of *Prunus persica* var. *nucipersica* (nectarine).

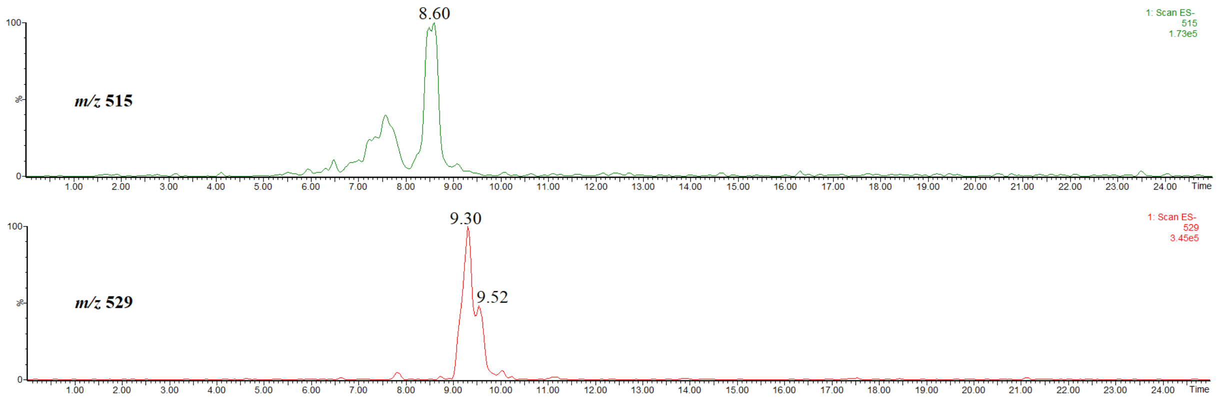

Figure S8. Single ion chromatogram of the ion at *m/z* 515 (the $[M-H]^-$ ion of 3,5-di-*O*-caffeoylquinic acid) and *m/z* 529 (the $[M-H]^-$ ion of methyl 3,5-di-*O*-caffeoylquinates) obtained for the extracts of kernels of *Prunus persica* var. *nucipersica* (nectarine).

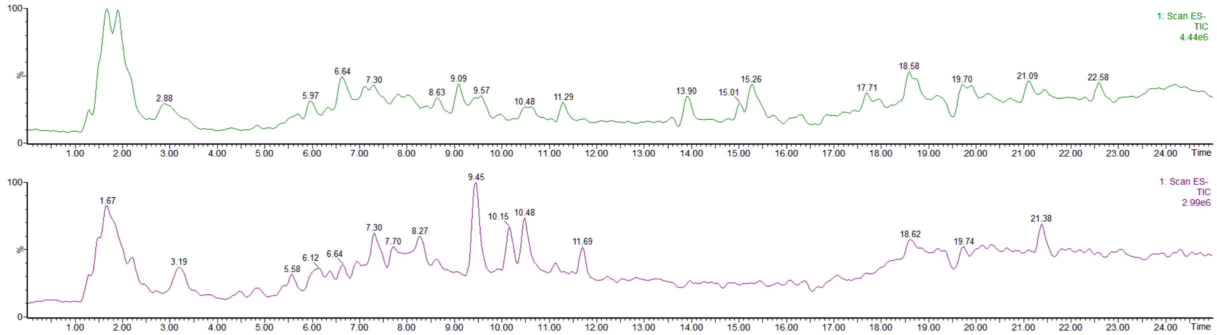

Figure S9. Total ion current chromatograms obtained for the extracts of *Crataegus monogyna* inflorescences by using methanol (top) and acidified methanol (bottom).

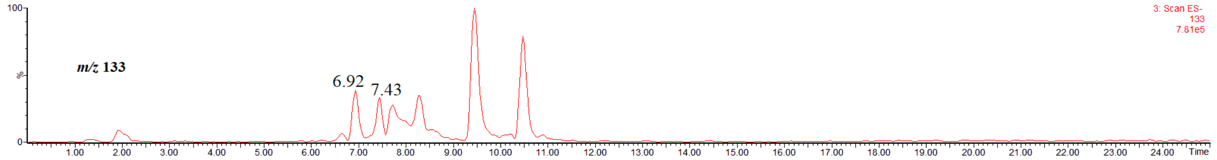

Figure S10. Single ion chromatogram of the ion at *m/z* 133. The peaks at rt 6.92 and 7.43 min clearly indicate that product ions at *m/z* 133 originates from methyl 3-*O*-caffeoylquinate and methyl 4-*O*-caffeoylquinate – see Figure 4 in the manuscript.
